# Moving Diabetes Prevention Programs to the Workplace: A Qualitative Exploration of Barriers and Facilitators to Participant Engagement When Implemented by an Employer-Based Clinic

**DOI:** 10.5888/pcd21.240173

**Published:** 2024-10-24

**Authors:** Sandra A. Tsai, Alexandria Blacker, Jonathan G. Shaw, Cati Brown-Johnson

**Affiliations:** 1 Division of Primary Care and Population Health, Department of Medicine, Stanford University School of Medicine, Stanford, California

## Abstract

**Purpose and Objectives:**

The Diabetes Prevention Program (DPP), an effective evidence-based strategy to reduce the incidence of type 2 diabetes, has been widely implemented in various locations, including workplaces. However, most people do not remain engaged in the program for the recommended full year. Limited qualitative research exists around participant engagement in the workplace DPP. Our study aimed to explore participant engagement in the DPP delivered through the employer-based clinic (EBC) at a large technology company.

**Intervention Approach:**

The DPP was implemented through the EBC at a large technology company in Southern California, beginning in September 2019 by using in-person and virtual synchronous group classes before and during the COVID-19 pandemic.

**Evaluation Methods:**

Virtual focus groups with DPP participants from 2 inaugural cohorts were conducted via Zoom from October 2020 to February 2021. Data were analyzed by using inductive thematic analysis.

**Results:**

Five focus groups with 2 to 3 participants in each (total n = 12) were conducted, 2 focus groups per cohort and 1 focus group with the group instructors. Barriers and facilitators to engagement in the DPP were grouped into thematic domains: Individual Drivers, Small Group Community, Workplace Setting, Integrated EBC, and the COVID-19 Pandemic. Results showed that prepandemic workplace demands (ie, meetings, travel) affected DPP participation, yet the group setting provided social support in the workplace to engage in and maintain healthy habits. With the move to a virtual synchronous offering during the pandemic, participants valued the group setting but expressed a preference for in-person meetings. Collectively, participant engagement was bolstered by shared buy-in and collaboration between the employer and the EBC.

**Implications for Public Health:**

Our findings suggest that engagement in a workplace DPP can be supported by addressing workplace-specific barriers and gaining buy-in from employers. Delivering the DPP, in person and virtually, through an EBC has the potential to engage employees who have prediabetes.

**Summary**

**What is already known on this topic?**

Most participants who enroll in the Diabetes Prevention Program (DPP) do not remain engaged for the recommended 12 months.

**What is added by this report?**

Delivering the DPP as a virtual, synchronous class through an integrated health care model of an employer-based clinic (EBC) reduced barriers to referrals from providers and facilitated participant employees’ engagement through the pandemic.

**What are the implications for public health practice?**

Using the EBC to deliver the DPP may be an important strategy in engagement for employee participants. Virtually delivered DPPs may play an important role with the increasing prevalence of hybrid work models, and they offer the potential to reach participants who cannot attend in-person classes.

## Introduction

The Diabetes Prevention Program (DPP) randomized clinical trial found that intensive lifestyle modification delivered in a year-long program reduced the incidence of type 2 diabetes by 58% among high-risk participants (1). The Centers for Disease Control and Prevention (CDC) created the National Diabetes Prevention Program in 2010 (2). Since then, the DPP has been widely implemented in approximately 1,500 different settings, including community centers, primary care clinics, churches, and worksites (3).

For people who enroll in the DPP to reap its full health benefits, they should ideally complete the full program. Recent studies had indicated that the degree of engagement, which prior authors defined as greater session attendance and more weekly physical activity minutes, predicted weight loss in community participants (4–7). In terms of longer-term benefits, the Diabetes Prevention Program Outcome Study, which followed participants from the original DPP trial for 15 years, found both lifestyle intervention and taking metformin reduced diabetes incidence by 27% (*P* < .001) and 18% (*P* = .001), respectively, compared with the control arm. In addition, in women (but not men), lifestyle intervention reduced microvascular disease by 21% (relative risk, 0.79) compared with placebo and by 22% (relative risk, 0.78) compared with metformin (8). However, evidence suggests that most people who enroll in DPP do not complete the course. Ely and colleagues explored high-intensity participation in the DPP, defined as completing 17 or more sessions (6). These authors found that among people enrolled from February 2012 to January 2016, only about 37% of enrollees met this threshold. Thus, better understanding of the barriers and facilitators of participant engagement is crucial to facilitate disseminating the DPP in ways that deliver its originally proven outcomes.

Recent evidence suggests that workplace DPPs are effective at preventing diabetes (4,9) and CDC has encouraged employers to play a critical role in helping employees prevent diabetes and cardiometabolic disease (10). Large employers are increasingly investing in employer-based clinics (EBCs) to enhance employee well-being, reduce health care costs, and improve productivity (11,12). According to the Business Group on Health, 53% of large employers invested in a worksite clinic in 2023; most are either occupational health clinics or primary care clinics (13,14). These clinics provide convenient access to primary care, preventive services, and occupational health services, reflecting a strategic focus on integrated health care management and employee health outcomes. Prior qualitative studies reporting on factors affecting attendance and engagement in DPP sessions are limited (11–14). Notably, a significant gap exists in the literature regarding qualitative studies reporting facilitators and barriers to participant engagement in a workplace DPP, particularly those delivered within an EBC. Our research aims to fill this gap.

## Purpose and Objectives

The objective of our implementation study was to explore barriers and facilitators to participant engagement in a workplace DPP delivered through an EBC and to examine how converting from an in-person to virtual delivery mode during the COVID-19 pandemic affected engagement. We defined engagement as enrolling in the DPP, attending and participating in classes, and doing class activities, such as measuring one’s weight, being physically active, and eating healthily. Our study aimed to explore participant engagement during the DPP, not after the program ends. We used qualitative methods to evaluate engagement in the DPP and to highlight key learnings for future implementation in similar settings.

## Intervention Approach

An academic–corporate partnered EBC at a large technology company in Southern California implemented the DPP for its employees in September 2019. The EBC is located on the company’s campus and provides comprehensive primary care services and on-site chiropractic care, physical therapy, optometry, and behavioral health services. The clinic is independently operated by Stanford Health Care, with physician staffing and leadership provided by the Stanford School of Medicine.

The DPP lifestyle change program consists of weekly classes for 2 months, semiweekly classes for 4 months, then monthly classes for 6 months for a total of 22 class sessions. Our first cohort (Cohort 1) started September 2019 and the second cohort (Cohort 2) started March 2020 (Figure). Cohort 1 classes began as in-person sessions, then moved to virtual synchronous sessions after 6 months at the start of the COVID-19 pandemic. Cohort 2 classes were exclusively virtual synchronous sessions because of the pandemic’s shelter-in-place restrictions. Both cohorts were led by the same group instructors. One instructor was a registered dietitian, and one was a population health registered nurse. Employees of the technology company were eligible for the DPP if they had prediabetes and received care at the EBC. The DPP group instructors used the electronic health record patient portal to invite patients with a diagnosis of prediabetes to join the DPP. The instructors identified potential participants by running an automated report in the electronic medical record to find patients with HbA_1c_ levels in the prediabetes range (5.7%–6.4%) and sent them a bulk, nonpersonalized message about the program. Employees were made aware that there was no cost to participate in the DPP. They also learned about the DPP through their EBC primary care team, which made direct referrals to the program. The EBC physicians learned about the DPP through a presentation at their monthly staff meeting and individual outreach from the DPP group instructors.

**Figure:**
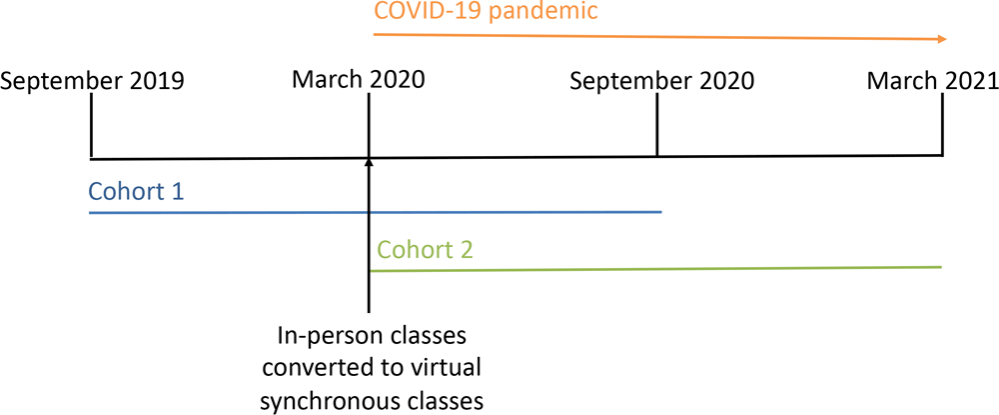
Timeline for the Diabetes Prevention Program, Cohort 1 and Cohort 2, implemented by an employer-based clinic. The first cohort (Cohort 1) started in September 2019 and the second cohort (Cohort 2) started in March 2020.

## Evaluation Methods

### Design

For our study, we invited DPP participants from the 2 inaugural cohorts and their group instructors to join focus groups to describe perceived barriers and facilitators to participant engagement. An exploratory qualitative study approach was used to explore themes (15), and the qualitative data were collected via virtual focus groups.

### Selection and recruitment of participants

A convenience sampling technique was used to recruit focus group participants from the 2 cohorts who had recently completed or were about to complete the DPP. Cohort 1 had 14 participants (4 women) and Cohort 2 had 12 participants (2 women). An invitation to participate in a 1-hour focus group was sent via email to members of both cohorts, and a $25 DoorDash gift certificate was given to thank them for their participation.

### Focus groups

We conducted 5 focus groups with a total of 12 participants (2–3 participants in each): 2 focus groups for cohort 1; 2 focus groups for cohort 2; and 1 focus group with the group instructors. Five members from each cohort participated in the focus groups along with 2 group instructors. Because of pandemic restrictions, the focus groups were video- and audio-recorded via Zoom with the permission of the participants and lasted from 45 to 60 minutes. The groups were led under the supervision of a PhD-trained qualitative researcher (C.B.J.) and conducted between October 2020 and February 2021, with the support of two MPH-trained colleagues, a doctoral student (A.B.) and a physician (S.T.), with participants who had recently completed or who were about to complete the DPP. All participants resided in California at the time of the focus group. Group discussion used a semistructured interview guide with open-ended questions relating to barriers and facilitators to engagement, which included outreach, enrollment, participation in the course activities, and meeting format. Two semistructured interview guides were use, 1 for group facilitators (Table 1) and one for DPP participants (Table 2).

**Table 1:** Instructor Semistructured Interview Guide, Diabetes Prevention Program (DPP) Group

Interview domains	Questions
Outreach	How did referred patients learn out about the DPP?
Outreach	What role has [company] played in making the DPP available to its employees?
Barriers to enrollment	For people who were referred to the program but not enrolled, why do you think they did not join the program?
Barriers to engagement	Throughout the program, what reasons prevented people from attending in-person classes? Video classes?
Facilitators of engagement	Throughout the program, what supported the participants’ ability to change and sustain recommended behaviors?
Barriers to engagement	What factors made participants’ ability to change and sustain recommended behaviors difficult?
Facilitators of engagement	What factors would have made participants’ ability to report their physical activity, diet, and weight easier?
Barriers to engagement	What factors made participants’ ability to report their physical activity, diet, or weight difficult?
Facilitators to engagement	Throughout the program, from recruitment to the end of the program, what factors motivated people to engage in the program?
Facilitators to engagement	What would you like to change to increase participant involvement and participation throughout the program, from recruitment to the end of the program?
Meeting format	How was the experience for the group and for the facilitators when the DPP went from in person to virtual?
Meeting format	What were the positives and negatives about doing the DPP in person versus virtual as it relates to participant involvement in attendance to sessions and behavior change?

**Table 2:** Participant Semistructured Interview Guide, Diabetes Prevention Program (DPP)

Interview domains	Question
Outreach	How did you learn about the Stanford DPP?
Outreach	What role has [company] played in your participation in the Stanford DPP?
Facilitators to enrollment	What motivated you to join the Stanford DPP?
Facilitator to engagement	How do you overcome factors that make it difficult for you to make it to class?
Barrier to engagement	What factors make it difficult for you to make it to class? (followup: Did this differ for in-person versus video?)
Facilitator to engagement	How do you overcome factors that make it difficult for you to make it to class?
Facilitators to engagement	What changes to the program would you recommend to make it easier to come to class?
Facilitator to engagement	What factors support your ability to change and sustain the behavior changes that you’ve learned in class?
Barriers to engagement	What factors most get in the way of your ability to change and sustain behaviors learned in class and to continue with the yearlong program?
Facilitator to engagement	What factors would make it easier to report your physical activity, diet, and weight?
Barrier to engagement	What, if any, factors made it difficult to report your physical activity, diet, or weight?
Meeting format	What do you like about meeting in person?
Meeting format	What would you change about meeting in person?
Meeting format	What do you like about meeting virtually?
Meeting format	What would you change about meeting virtually?
Meeting format	Thinking about the entire Stanford DPP program, what format would you prefer the classes be delivered in? For example, are there parts of the program/series of classes that would be better for one or the other?
Participation	What recommendations do you have to make this program more accessible to your peers who did not participate?
Participation	What would you tell a work colleague or friend who was considering the program?
Participation	What would you suggest change to increase recruitment and participation among your peers?

### Data analysis

Focus group discussions were transcribed from the Zoom recording and all transcripts were coded in NVivo 1.4.1 (Lumivero). Data were analyzed by using inductive thematic analysis (16). Three reviewers (C.B.J, S.T., A.B.C.) were involved in qualitative coding and analysis. Two reviewers double-coded 1 focus group transcript to develop a preliminary coding schema. Once a coding schema was developed, focus group transcripts were coded individually. Coding was reviewed by all 3 members by meeting regularly to reach consensus; changes were made to the codebook as necessary. The coded data were inductively examined for themes that represented perceived barriers and facilitators to participant engagement in the DPP. We found no thematic differences between the cohorts or group instructor focus groups, so data are presented in aggregate.

## Results

The DPP participant focus group consisted of 10 people. Their average age was 45 years; 4 participants were female, which was higher than the number of females (23%) overall in the DPP, and were of the following races or ethnicities: 30% White, 50% Asian, and 20% Hispanic. The instructor focus group consisted of 2 White females with an average age of 55 years. We refer to each participant (P) by assigned numbers (eg, P1, P2) and instructors (I) by assigned number (I1 or I2).

Based on the inductive coding process, 5 themes emerged, and barriers and facilitators to participant engagement were identified for each. The 5 themes were individual drivers, small group community, workplace setting, integrated EBC, and COVID-19 pandemic. We described the detailed results for each domain and the barriers and facilitators to engagement of each theme (Table 3).

**Table 3:** Summary of Barriers and Facilitators to Participant Engagement in Diabetes Prevention Program, by Theme

Theme	Barrier	Facilitator
Individual drivers	Limited bandwidth and motivation, especially during COVID-19	Knowing their diabetes risk and the perceived benefits of the Diabetes Prevention Program
Small group community	Challenge of virtual social support, compared with in-person	Effective instructors and sharing with others who have similar struggles
Workplace setting	Competing demands at work	On-site DPP meetings and resources
Integrated employer-based clinic	None mentioned	Ease of access to health care services
COVID-19 Pandemic	Pandemic magnified barriers to healthy behaviors	Adjustment to pandemic life

### Theme 1. Individual drivers

**Barrier: Limited bandwidth and motivation, especially during COVID-19. **Participants said life was busy, which made engaging with the DPP difficult. DPP instructors observed that employees’ significant work demands and stress could make finding time to make healthy lifestyle changes difficult. One instructor (I2) said that “workload and stress levels are very, very high.” This then played into (I2) “their ability to find the time to exercise, to find the time to do meditation or relaxation.” Participants echoed this, with one (P1) saying “sometimes it was just too many things to do.”

During the COVID-19 pandemic, personal accountability became more important than external accountability because of social isolation. Participants said that being accountable to others could bring up feelings of shame if their lifestyle behaviors had not improved. They noted that making behavior change ultimately required having enough self-motivation.

. . . the problem comes up if I am self-motivated enough to sustain it for a long time . . . I think my longest was about three months. But then, you know up and down . . . especially when there's a lot of work, just end up binge eating or something like that. (P9)

**Facilitator: Knowing diabetes risk and the perceived benefits of the DPP. **Learning about their risk for diabetes and how to prevent it motivated participants to engage in healthy changes. Increasing knowledge about a healthy diet, exercise, and weight maintenance supported participants in making practical lifestyle changes. For one participant (P4), knowing they could change the course of getting diabetes by making healthy changes made joining the DPP seem obvious: “I have to do everything that I can to stop this [diabetes] from occurring to me.” Another participant said that their family history of diabetes pushed them to be proactive to avoid it.

Participants reported that the DPP’s inclusion of tracking (ie, participant’s food intake, physical activity, body weight) and regularly scheduled meetings helped to enhance their knowledge about how to put healthy behavior change into practice. Several participants mentioned the importance of this accountability, highlighting that the group acted, “ . . . like a nudge to improve your activity numbers.” (P6).

Knowing that others would learn about their progress encouraged participants to make behavior changes.

Accountability, going to this meeting and saying, “Hey look I really worked on myself for the next four weeks, and this is where I stand,” that was for me, the main reason [for making healthy behavior change]. (P8).

### Theme 2. Small group community

**Barrier: Challenge of virtual social support, compared with in-person meetings. **Participants said community building was more difficult with virtual meetings than with in-person meetings. Participants from both Cohort 1 and Cohort 2 expressed a preference for in-person meetings because the social interaction was more intimate. One participant from Cohort 2 said that having only the option to meet virtually likely stymied the group sharing dynamic.

. . . because everybody is kind of a little bit shy and doesn’t know what to say and everybody stands on a different level for this class so I think it would have probably given some people more support and an exchange to be a little bit more open about you know where they come from and what they are struggling with. (P8)

For those participants who started with in-person meetings, shifting to virtual meetings was better than not meeting, but in-person meetings ultimately were preferred. Sharing was not as seamless on video.

. . . we are on video and that is somewhat uncomfortable, but when we meet in person . . . it’s like friendly and warm and we are able to share anything even in our normal day routine if it’s something or we cannot do something, all those information which is like little bit restricted when we move to videos. (P2)

**Facilitator: Effective instructors and sharing with others who have similar struggles. **The group format of the DPP facilitated sharing and learning for both in-person and virtual settings. Participants talked about the importance of hearing about common struggles, using the group dynamic to solve problems, and the group becoming a supportive environment. Participants felt accountable to the group and believed the group could help push them to continue building healthy habits.

[It] was just to get on track and being in a group, I think, is beneficial. You hear from other people having successes or is just pushing yourself a little bit (P1).

The group instructors were pleased to see the level of engagement of Cohort 1.

But I really think the group was just especially [engaged] because we’re all in-person and they really were just engaged . . . we were worried that nobody was going to show up and all of a sudden, we have this room full of people, and it was a party, and they were sharing . . . some of them getting teary (I1).

The group instructors also said creating group support and enhancing the group dynamic was important. One instructor said that as participants progressed with the group, they increased self-efficacy in adopting healthy behaviors and a positive mindset.

[They] learned something along the way. So always looking at the positive and I think we’ve heard that from people: “I know I have the tools. I know what to do” (I1).

Participants uniformly agreed that their instructors were instrumental in program engagement.

I think that we couldn’t have picked two better teachers. I think they’re very compassionate, they listen. They know where we’re at, they know what we’re trying to accomplish. They reinforce things. Yeah. So, it was a great class (P3).

### Theme 3. Workplace setting

**Barrier: Competing demands at work. **Participants noted that a major barrier to attending the workplace DPP meetings was competing workplace demands, particularly overlapping meetings. Before the pandemic, the in-person classes were scheduled to meet at the worksite over lunch. This took place weekly for 4 months, biweekly for 2 months, then monthly for 6 months. Several participants said a demanding workload, which potentially could include significant travel, sometimes conflicted with the DPP meeting times. Work meetings overlapping with the DPP meeting was especially problematic for participants who did not proactively block off their work schedules for DPP.

. . . [I] take measures such as blocking out [my calendar] and deliberately cutting [the] other meeting short to make room for it, but it worked out well that Thursday was somewhat less contested during the course (P7).

During the pandemic, the workload seemed to increase because the expectation from coworkers was that everyone would be online continuously without set breaks for lunch, which conflicted with the DPP meetings.

I think just the norm that people expect, you know, “Hey, you're at home, you're available, you're just sitting there. We're going to do a lunch hour meeting,” because everyone's available during lunch hours (P3).

**Facilitator: On-site DPP meetings and resources.** Prepandemic, workplace on-site meetings contributed to an increase in meeting attendance because of their close proximity and allowed people to participate during the workday even if their schedules became busy.

I used to have some meetings right before [the DPP class] and that meeting was always running late . . . I decided that it's better to join even 10 minutes late than not join and that seemed to work for me (P1).

The workplace site for this DPP included a free on-site gym, and prepandemic, some participants took advantage of this convenience by exercising at work. The DPP group instructors organized personal training sessions at the on-site gym for participants. This relationship facilitated exercise (at least prepandemic).

. . . everybody is very supportive of everybody's time and the ability and flexibility to take some time to walk over to [the gym] (P4).

One group instructor commented that the workplace environment was conducive to bringing employees together for a shared concern such as diabetes prevention, even when they did not know each other initially.

We had never really started group programs because of the concern that they [employees] may not really . . . feel comfortable, like in a group setting talking about things . . . which is understandable right in a corporate environment anyway? But . . . what we've seen . . . bringing them together and facilitating it in the right way and having that commonality, it actually is just exactly what they want and then what they need (I2).

### Theme 4. Integrated employer-based clinic

**Barriers: None mentioned.**

**Facilitator: Ease of access to health care services. **Because of the integration of the health care system into the workplace, employees could receive their primary care from the EBC and be referred by their primary care provider to the DPP, which was delivered through the EBC. Through the EBC, participants had annual check-ups and laboratory work that revealed prediabetes. This deemed them eligible to enroll in the DPP. Participants noted that when they learned they had prediabetes, it was motivating to be recommended to the DPP by their dietitian or primary care provider.

And then we got my numbers back . . . I freaked out . . . [my primary care provider] gave me more information. And she says, I think you're a good candidate for this [DPP] program (P4).

I’m just glad Stanford is there at our facility; it makes it so convenient (P3).

Furthermore, participants noted that receiving this information from their EBC clinician was motivating. One participant said that they “did not want to start any kind of medication and wanted to get it [the prediabetes] under control.” (P8)

### Theme 5. COVID-19 pandemic

**Barrier: The pandemic magnified barriers to healthy behaviors. **The COVID-19 pandemic acted as a multiplier to many of the previously described barriers. For instance, work hours increased, and work and home life became more stressful, leading to participants falling back into unhealthy habits.

In both Cohorts 1 and 2, the onset of the pandemic caused a rapid shift in participant priorities. Lockdown measures caused their daily environment to shift from the workplace to home. Additionally, concerns around infection and safety increased psychological distress. The focus on preventing diabetes became less important, with one participant noting that, “when the lockdown started somehow my priority shifted to other aspects. And it was a little bit more difficult.” (P1)

Additionally, some participants said that at the beginning of the pandemic they exercised less, ate less healthily, and gained back their weight.

When we started staying at home, and were not allowed to go out, I actually gained almost what I started [with] . . . we have restriction in what food items we can get online and all those things. So yeah, during that time I gained a lot of weight . . . (P2)

The challenges of adjusting to working from home and personal losses from COVID-19 increased stress among participants. Participants also said that because they were homebound, they were unable to get the daily exercise that they maintained at work and to stay consistent with their health goals.

From a health perspective, it was not healthy. I was not as active. At work I bounced between the different buildings. So, I would walk to all my meetings. Whereas here, you know, you get out of bed, in your day pajamas and you're sitting, sitting there from 7 to 5. (P3)

**Facilitator: Adjustment to pandemic life. **Participants reported that they reverted at first to previous poor health habits, but as they adjusted to life in lockdown, many said that they were able to make the best of a bad situation. Because travel for work stopped, participants were able to focus on a continuous, healthy meal plan that was uninterrupted by air travel and constant meetings. They were able to readily visit with their primary care provider in a virtual setting that was less disruptive to their work schedule. Additionally, participants said that working from home afforded increased flexibility to find time to be physically active.

When you’re at work that prep time [to workout] has to be done somewhere. I can’t be in a meeting and be changing or shampooing. So that's the reason it's tougher to do [workouts] at work. (P6)

## Implications for Public Health

Understanding barriers and facilitators to participant engagement in the DPP is crucial for optimizing program efficacy and assisting participants in maximizing its benefits. Our study found that both the workplace setting and the integrated EBC health care system were strong facilitators for participant engagement, and the virtual synchronous class led by engaging group leaders supported group cohesion during the pandemic, However, participants expressed a preference to meet in person for their group class.

As in previous research, we found that the group instructors’ interpersonal and facilitation skills were an integral piece of the group’s cohesion and an important contributor to participant engagement in the DPP (17). Although the employees of this large technology company were unknown to each other at the start of the DPP, they had no trouble connecting. Furthermore, in alignment with existing DPP literature, group support from fellow employees was integral to participant engagement, accountability, and maintenance of behavior change (17,18). We found that this trend was maintained even when the course was delivered virtually. Additionally, our study points to the importance of individual motivation. We found that a participant’s prediabetes status and a family history of diabetes were important motivational factors to engage in the DPP and support behavior change efforts. When individual motivation was low, such as when someone did not meet their weight loss goals, group support became ever more important.

We found specific facilitators and barriers related to the workplace setting that suggest that employers have a unique opportunity to play an integral role in participant engagement in a workplace DPP. Participants believed having their employer support the DPP by making it available to them without cost, having an on-site gym, and having on-site DPP meetings over the lunch hour were facilitators to engagement. Competing workplace demands (eg, meetings, travel) were barriers. Such barriers noted in prior qualitative research on community DPPs, including cost, location, meeting time, and conflicts with work schedule (19), could be removed by having an affordable workplace DPP. Prior research suggests that some workplace DPPs are less effective than others because of workplace characteristics, such as the social and physical environments (9). Thus, when considering the implementation of a workplace DPP, employers should consider how to integrate the program within the organizational infrastructure, such as scheduling meetings at a time when most employees can attend and coordinating with on-site or nearby fitness facilities. Modifying workplace demands can be challenging for employers, but prioritizing employee health warrants their diligent consideration throughout program implementation.

Our study also showed the advantage of delivering the DPP through an integrated EBC model, which facilitated communication among everyone involved. Because of the integrated model, DPP group instructors could reach out to primary care providers who learned the DPP was available to their patients. If a patient was interested in joining, the clinician could refer them to the DPP in the electronic health record and follow the patient’s progress. Previous research on barriers to referral found lack of clinician knowledge about the DPP to be a common barrier (19). Because primary care providers can often motivate their patients to participate in the DPP, the EBC model underscores the benefit of implementing a DPP within an integrated employee health care model (18,20).

Having the option to participate virtually during the pandemic was a facilitator for engagement. Although the DPP was originally implemented as an in-person program, nearly 250 DPPs are exclusively distance learning (3). Virtually delivered DPPs are crucial because of the increasing prevalence of hybrid work models, which necessitate flexible and accessible health interventions that can accommodate employees working both remotely and on site. Virtual programs are important because they offer the potential to reach participants who cannot attend in-person classes; however, not all virtual DPPs successfully engage participants. A large multistate study exploring engagement in virtual versus in-person DPPs found that people referred to an online DPP were more likely to enroll, but less likely to remain engaged in the program (21). An online DPP may be convenient, but leaving it may be just as easy, which underscores the importance of developing strong group cohesion to motivate participants. Thus, workplace DPPs may have the advantage of creating a shared workplace identity among participants, which may bolster group cohesion and participant motivation.

Our study had several limitations. First, only those who enrolled in the DPP were eligible for the study, because its aim was to explore engagement during the DPP. Further examination of the barriers and facilitators to enrolling in the DPP for similar populations is warranted. Additionally, we collected limited demographic information to assure confidentiality in the workplace setting. Future studies may benefit from exploring barriers and facilitators to participant engagement based on demographic characteristics and identifying any similarities or differences. Finally, we did not collect data segmented on duration of participation in the DPP. Subsequent work would benefit from a deeper understanding of the various barriers and facilitators to participant engagement based on length of time in the program, as well as longer-term assessment of experience and sustained behavior changes of participants after completing the workplace DPP.

In summary, our qualitative study found that a workplace DPP delivered through an integrated EBC affected employee participant engagement. Participant engagement in turn was affected by competing workplace and life-directed demands, but personal motivation, group support, and accountability promoted program engagement. The virtual synchronous class option was important and appreciated during the pandemic, but incorporating in-person sessions during the year-long DPP may be needed for community building and group sharing. Delivering the DPP through an EBC fostered a sense of support from the employer, promoted an integrated approach to employee wellness, and reduced barriers to clinician referral to the DPP. Because our focus groups were conducted in only one EBC setting — a technology company with a largely young, majority Asian male population — our findings may not be applicable to other workplaces. Future research should explore the use of the DPP across diverse workplace settings with integrated primary care and examine how employers can support DPP implementation and employee engagement.
